# Editorial: Combination immunotherapy and immune response assessment of brain tumours

**DOI:** 10.3389/fimmu.2025.1639173

**Published:** 2025-06-18

**Authors:** Haihui Jiang

**Affiliations:** ^1^ Department of Neurosurgery, Peking University Third Hospital, Peking University, Beijing, China; ^2^ Center for Precision Neurosurgery and Oncology of Peking University Health Science Center, Beijing, China

**Keywords:** brain tumour, gliomas, brain metastases, immunotherapy, immune response

Brain tumours, particularly aggressive primary malignancies such as glioblastoma (GBM) and secondary metastases, represent a devastating challenge in oncology. Despite decades of research and the application of multimodal treatments involving maximal safe resection, radiotherapy, and chemotherapy, clinical outcomes remain dismal, with median survival often measured in months (1, 2). Immunotherapy, which has transformed the treatment landscape for several haematological malignancies and solid tumours through approaches such as immune checkpoint inhibitors (ICIs), cancer vaccines, adoptive cell therapies including CAR-T cells, and oncolytic viruses, has demonstrated limited efficacy in brain tumours (3).

Among these strategies, ICIs have shown disappointing results in unselected GBM cohorts, with most clinical trials failing to yield meaningful clinical benefit (4). This highlights the complex immune evasion mechanisms of brain tumours, which include T cell exhaustion, the enrichment of immunosuppressive immune cell populations, secretion of inhibitory cytokines, and the expression of immune checkpoint ligands. In response to these challenges, combination immunotherapy has gained increasing attention. Nevertheless, the complexity of multi-agent regimens presents substantial difficulties in clinical practice, such as managing overlapping toxicities, accurately evaluating treatment efficacy, and identifying patient subgroups most likely to benefit. Therefore, robust and dynamic immune response assessment has become essential for guiding treatment strategies and improving outcomes.

This Research Topic brings together nine contributions that examine immunotherapy and immune response evaluation in brain tumours from molecular, cellular, therapeutic, and clinical perspectives. Immune checkpoint inhibitors (ICIs) are currently the most mature and widely used form of immunotherapy. While high PD-L1 expression is often associated with better responses to ICIs in various tumours, this correlation has not been consistently observed in gliomas (5). Ni et al. reported that brain metastases with high PD-L1 expression can still benefit from ICIs, and Wu et al. described a case of long-term survival in a patient with non-small cell lung cancer (NSCLC) brain metastases exhibiting high PD-L1 levels. These findings suggest important biological and therapeutic differences between primary brain tumours and secondary brain metastases in their responsiveness to immunotherapy.

Beyond ICIs, novel immunotherapies such as cancer vaccines, oncolytic viruses, and CAR-T cell therapy have also been explored for treating brain tumours. Yang et al. reviewed recent advances in the immunotherapy of primary central nervous system lymphoma (PCNSL), highlighting the potential of combination strategies involving chemotherapy or targeted therapies to enhance efficacy. Similarly, the limited success of monotherapy in GBM reinforces the rationale for combinatorial approaches as a necessary step toward improving treatment outcomes (6).

Liu et al. presented a promising therapeutic regimen that combines low-dose radiotherapy with immunoadjuvant treatment in patients with recurrent GBM. Radiotherapy has the potential to induce immunogenic cell death, which leads to the release of tumour antigens and stimulates the host immune response, effectively generating an *in situ* vaccination effect (7). In certain cases, this may even elicit an abscopal effect, where localized radiation results in a systemic anti-tumour immune response (8).

While T cell activation, expansion, and reversal of exhaustion remain central goals of immunotherapy (9), increasing attention has been given to the roles of other immune cells, particularly dendritic cells (DCs) (10), and tumour-associated macrophages (TAMs) (11), in shaping the tumour immune microenvironment. Gardam et al. emphasized the importance of the DC–T cell axis and proposed strategies to enhance DC recruitment and function as a means to augment T cell-based immunotherapies. Ye et al. focused on the crosstalk between TAMs and glioma cells, demonstrating that IL4I1 promotes M2-like macrophage polarization and enhances glioma invasiveness. Their findings suggest IL4I1 as a promising therapeutic target for selectively reprogramming TAMs, thereby supporting the development of more precise immunotherapeutic interventions in gliomas.

A critical challenge in clinical practice is the identification of patients who are likely to benefit from immunotherapy and the accurate assessment of treatment efficacy. Biomarkers such as PD-L1 expression (Wu et al.; Ni et al.), high tumour mutational burden (Ni et al.), and microsatellite instability (Ni et al.) have been associated with favourable responses to ICIs in brain metastases. However, in GBM, higher tumour mutational burden may be associated with resistance to immunotherapy, as suggested by Liu et al., highlighting the need for context-specific biomarker interpretation. Yang et al. employed integrated multi-omics analyses to define disulfidptosis-based subtypes of GBM, providing new insights for personalized immunotherapy, targeted therapy, and chemotherapy selection.

At present, assessment of immunotherapy efficacy in brain tumours relies heavily on magnetic resonance imaging (MRI). However, due to the spatial and temporal heterogeneity of immune responses and the unique features of the central nervous system, conventional imaging often fails to capture the complexity of therapeutic effects (12, 13). Fortunately, the advent of artificial intelligence (AI) technologies offer transformative potential for decoding complex imaging biomarkers in neuro-oncology (Chen et al.). By applying deep learning architectures to multimodal MRI datasets, we can generate more objective and reproducible imaging biomarkers, enabling improved evaluation of immunotherapy responses beyond the limits of current qualitative assessment methods.

**Figure 1:**
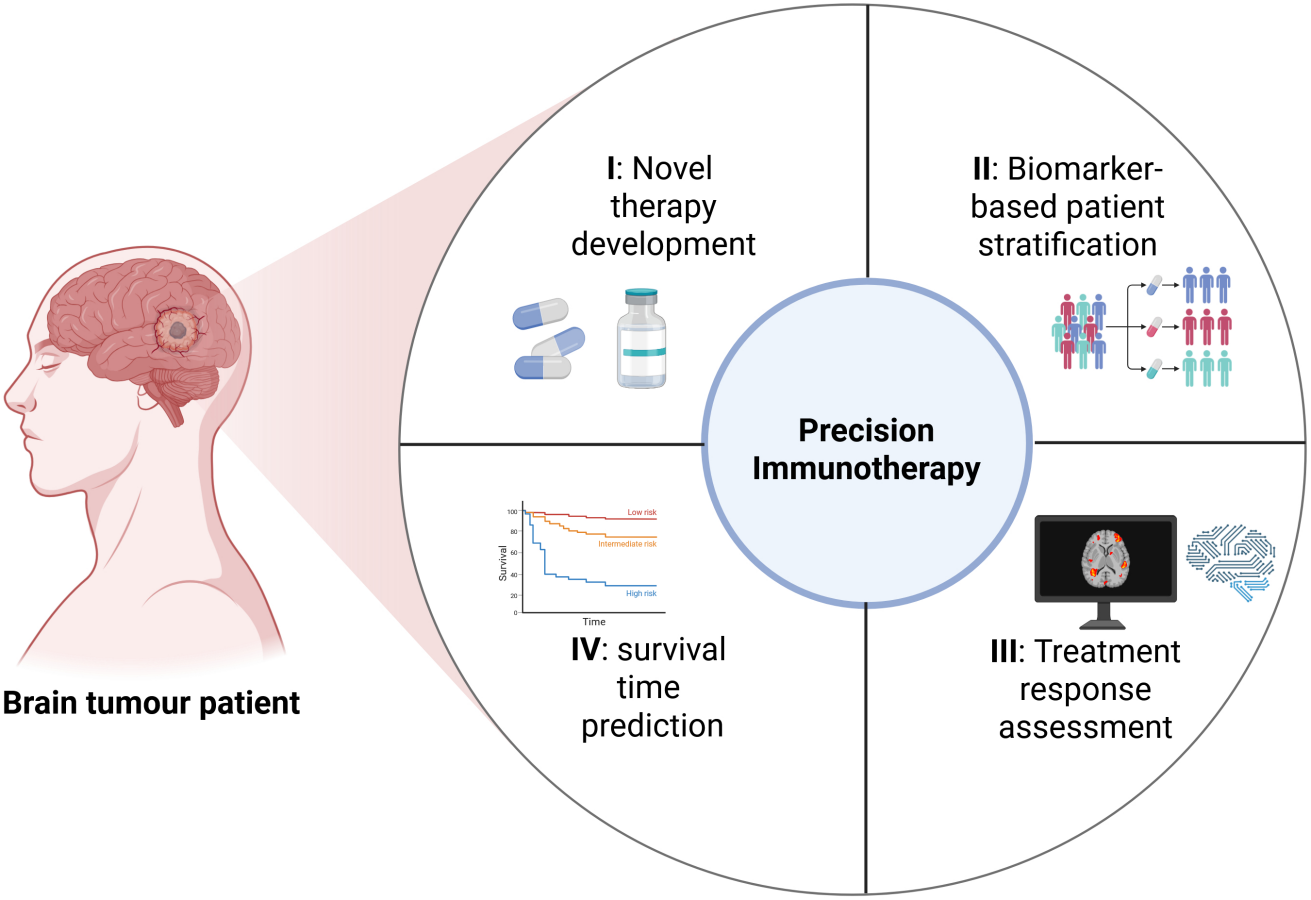
A comprehensive translational research framework that initiates with novel therapy development, biomarker-based patient stratification, treatment response assessment, and survival time prediction (Created with bioRender.com).

In conclusion, this Research Topic reflects the growing momentum toward precision immunotherapy for brain tumours. The evidence supports the integration of immunotherapy with radiotherapy, chemotherapy, and targeted agents to enhance therapeutic efficacy. Moreover, the application of AI-assisted image analysis and multi-omics integration is helping to identify the patients most likely to benefit from specific immunotherapeutic approaches. These advances are contributing to a comprehensive translational research framework that spans from novel therapy development and biomarker-based patient stratification to treatment response assessment and survival time prediction (Niu et al.) (**Figure 1**). Collectively, these efforts are propelling the evolution of personalized immunotherapy strategies for central nervous system malignancies and shaping the future of neuro-oncology.
